# Australian experience of a modified schedule of FOLFOX with high activity and tolerability and improved convenience in untreated metastatic colorectal cancer patients

**DOI:** 10.1038/sj.bjc.6602426

**Published:** 2005-03-01

**Authors:** D Goldstein, P Mitchell, M Michael, P Beale, M Friedlander, J Zalcberg, S White, S Clarke

**Affiliations:** 1 Department of Medical Oncology, Prince of Wales Hospital, Randwick, Sydney, NSW 2031, Australia; 2 Department of Medical Oncology, Austin and Repatriation Medical Centre, Melbourne, Australia; 3 Department of Medical Oncology, Peter MacCallum Cancer Centre, Melbourne, Australia; 4 Department of Medical Oncology, Royal Prince Alfred Hospital, Sydney, Australia

**Keywords:** colorectal cancer, dose intensity, 5-fluorouracil, neurotoxicity, oxaliplatin

## Abstract

This study determined the efficacy and safety of a modified FOLFOX regimen that improved patient convenience without compromising oxaliplatin dose intensity. A total of 62 patients with previously untreated metastatic colorectal cancer were enrolled to receive, entirely as outpatients, 2-weekly cycles of oxaliplatin 100 mg m^−2^ i.v. over 2 h, together with leucovorin 400 mg m^−2^ over 2 h, 5-fluorouracil (5-FU) 400 mg m^−2^, bolus, followed by a 46-h infusion of 5-FU at 2.4 g m^−2^. Treatment was given until progression or unmanageable toxicity. In all, 61 patients received ⩾one oxaliplatin dose and a median of 11 treatment cycles (range 1–20 cycles); 22 (36%) reported grade 3/4 neutropenia and 13 patients (21%) experienced grade 3 neurotoxicity; 16 patients (26%) discontinued treatment due to disease progression or death, 15 (25%) due to neurotoxicity and six (10%) due to haematological toxicity. Of the 56 eligible patients, complete or partial responses were observed in 29 or 52% (95% confidence interval 38–65%). Median progression-free survival was 8.2 months (7.1–9.9) and median overall survival was 18.7 months (14.0–23.4). In our experience, a modified schedule of FOLFOX improves convenience without compromising efficacy or toxicity.

Oxaliplatin unlike cisplatin has demonstrated activity against colon carcinoma cell lines *in vitro* and has also shown synergistic activity in experimental models (Raymond *et al*, 1997). Several large randomised trials have confirmed the efficacy of the oxaliplatin/5-fluorouracil (5-FU)/leucovorin (LV) combination as first-line therapy in metastatic colorectal cancer. In De Gramont's *et al* (2000) phase III trial, FOLFOX4 was significantly superior to the infusional regimen, LV5FU2, in response rate and progression-free survival (PFS) and the combination of chronomodulated 5-FU/LV and oxaliplatin produced a significantly higher response rate and superior PFS to the 5-FU/LV regimen alone (Giacchetti *et al*, 2000). In a recent three-arm study in 796 untreated patients with metastatic colorectal cancer (Goldberg *et al*, 2004), FOLFOX4 was significantly superior to the irinotecan and bolus 5-FU/LV combination (IFL) and a third regimen of oxaliplatin plus irinotecan (IROX) in response rate, time to progression and overall survival, and induced fewer Grade 3 or 4 toxicities.

The original FOLFOX4 regimen, however, does have drawbacks for patients, as it requires intravenous treatment in hospital for 48-h every 2 weeks. Several variations of this regimen, incorporating oxaliplatin dose intensification or simplification of the LV/5-FU schedule, have been evaluated in clinical trials.

A retrospective analysis by Maindrault-Goebel *et al* (2000) of three phase II studies, in previously treated patients, using three different FOLFOX regimens showed that increased oxaliplatin dose intensity (>85 mg m^−2^) improved response rates and PFS without compromising tolerability. One of the more effective regimens, FOLFOX6, utilised an oxaliplatin dose of 100 mg m^−2^ on day 1 in combination with a simplified bimonthly 5-FU/LV schedule (LV 400 mg m^−2^ on day 1 only, followed by 5-FU bolus 400 mg m^−2^, then 2400–3000 mg m^−2^ as a 46-h infusion). In the phase II trial with FOLFOX6 in pretreated patients, a response rate of 27% was achieved, but with significant grade 3 or 4 neutropenia (24%) and neurotoxicity of 16% (Maindrault-Goebel *et al*, 1999).

In this Australian phase II study in untreated patients with metastatic colorectal cancer, we also sought to simplify the treatment delivery schedule of the active FOLFOX4 regimen without compromising dose intensity. We eliminated the 2nd day bolus dose of 5-FU, increased the dose of oxaliplatin to 100 mg m^−2^ and increased the 5-FU infusion to 46 h, similar to FOLFOX6, and also utilised disposable pumps to allow an entirely outpatient treatment. In the algorithm for dose reductions due to haematological toxicity, we chose, in contrast to FOLFOX6, to preferentially dose-reduce 5-FU rather than oxaliplatin, aiming to maximise oxaliplatin dose intensity. In FOLFOX6 both the oxaliplatin dose was reduced to 75 mg m^−2^ and 5-FU infusion to 2000 mg m^−2^ without altering the bolus dose of 5-FU. In our schema the first dose adjustment was elimination of the bolus 5-FU, the second was reduction of the continuous infusion of 5FU and only then reduction of oxaliplatin. The primary objective of our study was to determine the objective response rate of this modified FOLFOX6 regimen. Our secondary objective was to assess the qualitative and quantitative toxicities of this regimen.

## PATIENTS AND METHODS

### Patients

Patients with a histologically confirmed diagnosis of advanced adenocarcinoma of the colon or rectum were eligible to be entered into the trial provided they were aged ⩾18 years, had measurable disease (defined as at least one lesion with at least one diameter ⩾2 cm), ECOG performance status (PS) of 0–2 and had completed adjuvant treatment 6 or more months prior to study entry. Patients who received prior adjuvant treatment with oxaliplatin were not eligible. To be included, patients also had to have a serum creatinine ⩽160 *μ*mol l^−1^, bilirubin ⩽1.5 × the upper limit of normal (ULN), ALT ⩽4 × ULN, an absolute neutrophil count (ANC) ⩾1.5 × 10^9^ l^−1^, platelets ⩾100 × 10^9^ l^−1^ and peripheral neuropathy ⩽NCI Common Toxicity Criteria (CTC) grade 1. Patients were required to provide signed informed consent.

Patients were excluded if they had received prior therapy for advanced colorectal cancer, any uncontrolled infection, a history of myocardial infarction within the previous 6 months or current clinical evidence of congestive heart failure or unstable angina, a history of any other cancer (except nonmelanoma skin cancer or carcinoma *in situ* of the cervix) unless in complete remission and off all therapy for that cancer for at least 5 years, central nervous system metastases, or were pregnant or lactating or could not provide informed consent. Men and women of reproductive potential had to agree to use effective contraception.

### Study design

The study protocol was approved by each of the participating institutions' ethics committees and was conducted according to the Declaration of Helsinki. This was an open-label multicentre trial to assess the response rate and quantitative and qualitative toxicities of a modified schedule of FOLFOX.

Patients were registered by the investigator faxing the registration form to the sponsor Sanofi∼Synthelabo and then treatment could commence.

### Chemotherapy

**Figure 1:**
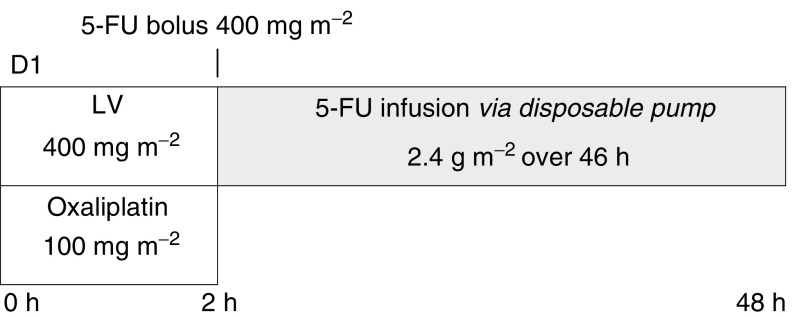
Chemotherapy regimen.

Treatment was given entirely as an outpatient. On day 1 of each 2-week cycle, patients received oxaliplatin (Eloxatin® supplied by Sanofi∼Synthelabo) 100 mg m^−2^ given as an intravenous infusion over 2 h together with LV 400 mg m^−2^ over 2 h and then 5-FU 400 mg m^−2^ as a bolus injection followed by a 46-h continuous infusion of 5-FU at a total dose of 2.4 g m^−2^ (Figure 1). Cycles were repeated at 2-week intervals until disease progression or unacceptable toxicity developed. Treatment with a 5HT_3_ receptor antagonist was recommended to control nausea and vomiting. All treatments were administered via implantable ports and using disposable pumps (Baxter Corporation).

### Dose modification

Toxicities were graded according to the NCI CTC version 2.0, 30 January 98, except for oxaliplatin-induced neurosensory toxicity, for which a specific grading scale was used. This scale was a combination of the NCI CTC and the Levi *et al* scale (Levi *et al*, 1992).

Doses of oxaliplatin and 5-FU were modified in a stepwise fashion according to a specific algorithm in the case of febrile neutropenia, ANC <0.5 × 10^9^ l^−1^, platelet nadir <50 × 10^9^ l^−1^ or a 1–3-week dose delay due to prolonged ANC, or platelet, recovery. Initially, the bolus dose of 5-FU was omitted from subsequent cycles; then, if the toxicity recurred, the 5-FU infusional dose was reduced to 2 g m^−2^ for subsequent cycles, and then the oxaliplatin dosage was reduced to 75 mg m^−2^ for subsequent cycles. Since 5-FU has previously been shown to contribute more to neutropaenia and since the bolus dose was the most convenient component to modify it was chosen first, followed by the infusional component. This strategy was subsequently also chosen by the French group in designing the Folfox7 regimen (Maindrault-Goebel *et al*, 2001). Treatment could be recommenced once febrile neutropenia resolved or the ANC improved to ⩾1.5 × 10^9^ l^−1^ or the platelet count improved to ⩾100 × 10^9^ l^−1^. If the toxicities recurred after the described 5-FU and oxaliplatin dose modifications, or a dose was delayed >2 weeks due to toxicity, study therapy was discontinued. No dose reductions were planned for changes in haemaglobin or total white blood cell concentrations.

Neurological symptoms were assessed according to a specific Neurosensory Toxicity Scale, which rated neurological symptoms Grade 1–4 according to their persistence, degree of functional impairment and impact on activities of daily living (ADL). Grade 1 described neurological symptoms that resolved and did not interfere with function, grade 2 symptoms interfered with function but not ADL, grade 3 toxicity indicated pain or functional impairment that interfered with ADL and grade 4 denoted persistent symptoms that were disabling or life threatening.

If patients experienced grade 2 or 3 neurosensory toxicity, oxaliplatin and 5-FU were both withheld until symptoms resolved and then restarted at a reduced dose of 75 mg m^−2^. If symptoms recurred, oxaliplatin was again withheld until resolution and then restarted at a reduced dose of 50 mg m^−2^. If symptoms recurred after two dose reductions, or persisted after a dose delay of >2 weeks, treatment was discontinued due to unacceptable neurosensory toxicity. Failure of neurotoxicity to return to grade I necessitated removal from the study. If pseudo-laryngopharyngeal dysesthesiae occurred, subsequent doses of oxaliplatin were administered as a 6-h infusion.

### Study parameters

Assessments for haematological parameters and biochemistry were performed every 2 weeks prior to each cycle and patients were questioned for symptoms of neurosensory toxicity prior to each oxaliplatin dose.

All tumours were measured by the same imaging method used at baseline for tumour size. A measurable tumour was defined as a mass with clearly defined margins and could be clearly measured on physical exam, X-ray or other radiograph with a ruler or caliper. At least one diameter had to be ⩾2 cm on radiographic imaging. Lesions with all dimensions <1 cm were omitted.

Responses were assessed every four cycles (8 weeks). Complete and partial responses were confirmed by repeat tumour evaluations at least 4 weeks later. Standard definitions and standard criteria for bidimensional measurement were used according to WHO (Miller *et al*, 1981) in assessing responses. Stable disease (SD) was confirmed after 4 weeks and had to last a minimum of 16 weeks from the commencement of therapy.

Progression-free survival was measured from the date of commencing protocol treatment to the date of first progression or death from any cause without progression. Overall survival was measured from the date of commencing protocol treatment to the date of death from any cause.

### Statistical considerations

Confidence intervals (CO) (95%) for the response rates were estimated using the exact probabilities of the binomial distribution.

The Kaplan–Meier method was used to calculate PFS and OS.

Statistical analyses were performed using StatXact procedures. (StatXact 4.0.1. Cytel Software Corporation, Cambridge, MA, USA, 1999).

Dose intensity was defined as the actual dose intensity relative to the protocol dose intensity. The initial doses of the drugs used were calculated using BSA in m2=exp (0.425 × ln(weight)+ 0.725 × ln(height)−4.9358) to assess whether initial doses were given as per protocol. The relative dose intensities were calculated for each drug by dividing the total dose given as a proportion of the planned protocol dose by the total number of days on treatment relative to protocol time. The protocol time was 14 days for each cycle given. The total number of days on treatment was calculated from day 1 of the first cycle to 14 days after day 1 of the last cycle.

## RESULTS

### Patient characteristics

A total of 62 patients were registered from four Australian centres between September 1999 and October 2000. In all, 61 patients received ⩾one dose of oxaliplatin and were analysed for treatment and safety results. Pretreatment characteristics of these patients are listed in Table 1 . While the median age was 61 years (range 31–76), 59% were aged 60 years or older, 21% were over 70, 97% were Caucasian.

Six of the 62 registered patients were deemed ineligible; one who had received adjuvant treatment less than 6 months prior to study entry, two with insufficient measurable disease, one who had received prior radiosensitising chemotherapy for advanced disease and two with laboratory parameters outside the eligible range on the days of treatment. The remaining 56 eligible patients were analysed for efficacy.

At the close-out date of 22 May 2003, the median potential follow-up time from the commencement of treatment was 37 months (range 31–44 months).

### Treatment given

In total, 587 cycles were given, with a range of 1–20 cycles and a median of 11 2-weekly cycles per patient.

Out of 61 patients, 58 received more than one cycle of therapy. Of these, 46 (79%) experienced a dose reduction of more than 10% relative to the dose in cycle 1 for at least one drug. In all, 26 patients (45%) had their oxaliplatin dose reduced and one patient (2%) had their leucovorin dose reduced by >10% on at least one cycle as an unspecified administrative error. A total of 44 patients (76%) had their 5-FU bolus dose and 27 (47%) had their 5-FU infusion dose reduced by >10% on at least one cycle.

Although the protocol specified 14 days between cycles, only eight patients (14%) received all their treatment cycles within ±2 days of the protocol 14-day period. In total, 136 cycles (23%) in 50 patients were delayed more than 2 days. Of these, 116 cycles (20%) were delayed for toxicity reasons.

### Objective tumour responses

Of the 56 eligible patients, the best response to treatment was assessed as a complete response (CR) in three patients (5%) and a partial response (PR) in 26 patients (46%), for an overall objective response rate of 52% (95% CI 38–65%). The full response data are shown in Table 2 .

### Progression-free survival (PFS)

**Figure 2:**
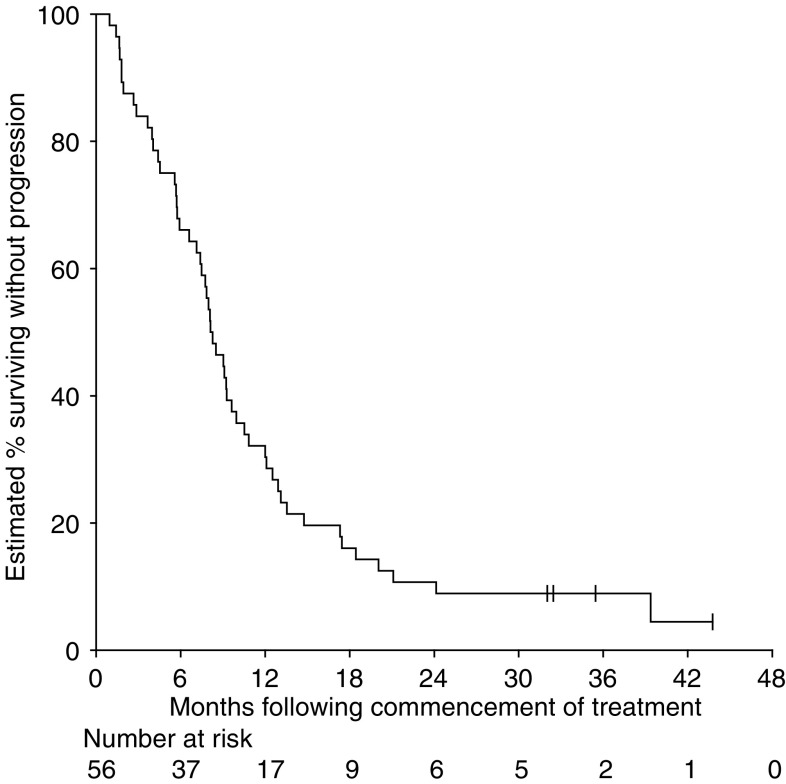
Progression-free survival. Ticks indicate censored survival times.

By the close-out date, four patients (7%) were alive without progression (two have since died) and 52 patients (93%) had progressed (two patients died without objective evaluation of their progression). The estimated median PFS was 8.2 months (95% CI 7.1–9.9 months) with an estimated proportion of patients surviving without progression of 30% (95% CI 20–44%) at 1 year and 11% (5–22%) at 2 years (Figure 2).

### Survival

**Figure 3:**
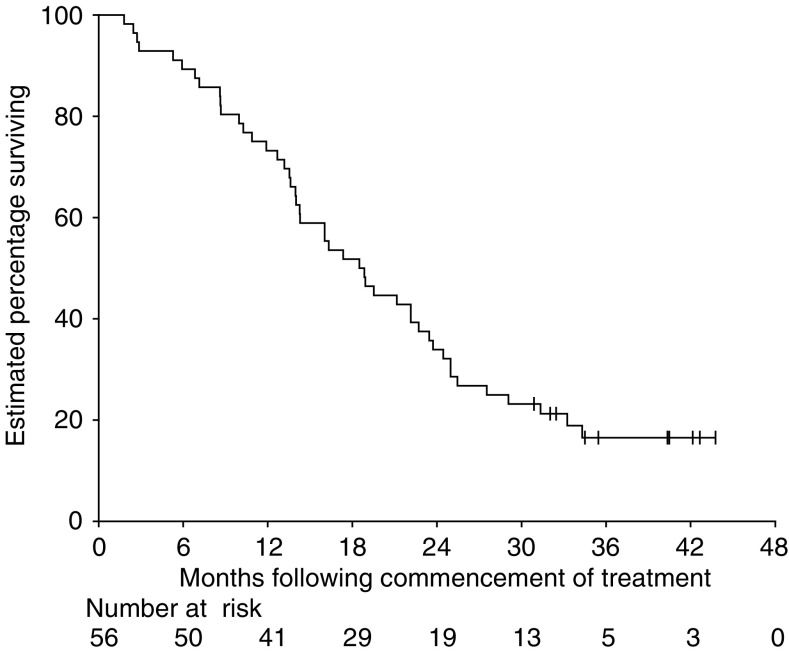
Overall survival. Ticks indicate censored survival times.

As at the close-out date, 10 (18%) patients were still alive and 46 (82%) patients had died, 45 (80%) due to progressive disease and one (2%) during surgery for complications of colorectal cancer. The estimated median OS was 18.7 months (95% CI 14.0–23.4 months), with an estimated proportion of patients surviving of 73% (95% CI 60–83%) at 1 year and 34% (23–47%) at 2 years (Figure 3).

### Dose intensity

**Figure 4:**
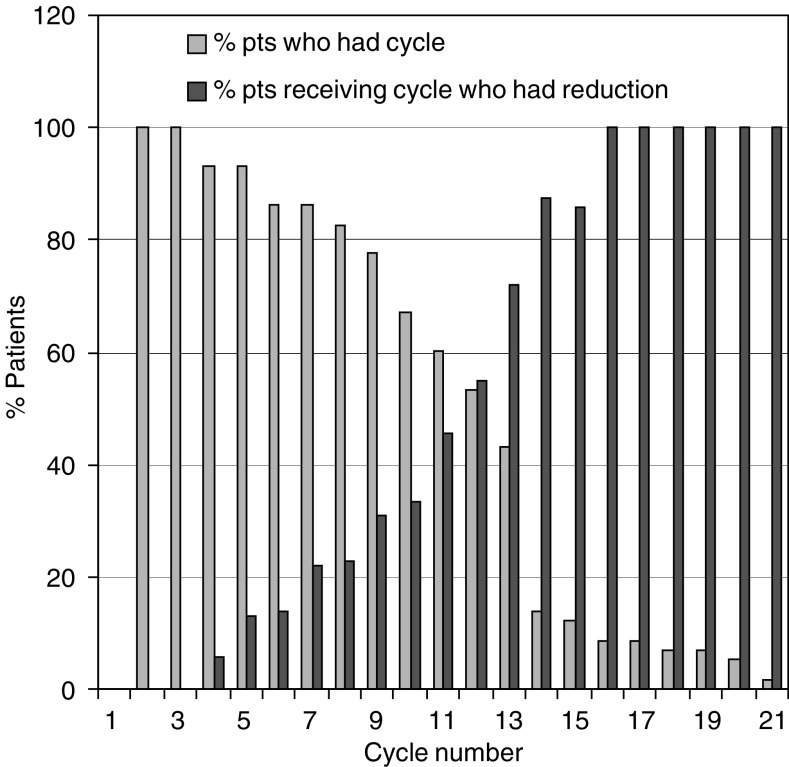
Dose intensity of oxaliplatin by cycle.

The median relative dose intensity of oxaliplatin was 83%, range 39–103%, of LV 88%, range 39–102%, of bolus 5-FU 50%, range 0–102% (omitted for haematologic or gastrointestinal toxicity) and of 5-FU infusion 83%, range 35–103%. In all, 55% of patients received 100+/5% of oxaliplatin for all cycles given – see Figure 4.

### Toxicity

At the study close-out date, of the 61 patients who received ⩾one treatment cycle, two patients (3%) had completed 12 treatment cycles and 59 patients (97%) had discontinued treatment. In all, 16 patients (26%) discontinued due to disease progression or death, 25 (41%) due to toxicity (15 (25%) neurotoxicity, 6 (10%) haematological toxicity and four (7%) due to other toxicity), 14 (23%) due to a decision of the patient or investigator and four (7%) due to other reasons.

Grade 2, 3 and 4 acute toxicities are listed in Tables 3 and 4 . The most commonly reported toxicity was neutropenia. Grade 3/4 neutropenia occurred in 22 (36%) patients; however, only three patients (5%) experienced febrile neutropenia. There were no toxic deaths.

Neurological toxicity was also common with 20 patients (33%) experiencing grade 2 and 13 patients (21%) experiencing grade 3 neurotoxicity during or post treatment. No grade 4 neurotoxicity was reported.

For patients with grade 2/3 neurotoxicity, the median time to recovery to grade 0/1 after ceasing treatment was estimated via the Kaplan–Meier method to be 3.9 months (95% CI 0–6.7 months). It was also estimated that 36% (21–54%) and 21% (10–40%) had persisting grade 2 or 3 neurotoxicity at 6 and 12 months, respectively. For grade 3 neurotoxicity specifically, it was estimated that 71% of the patients had recovered 12 months after ceasing treatment.

Two patients experienced a worsening of their neurological toxicity from grade 1 to 2 and one patient reported grade 3 myopathy after ceasing treatment.

### Hospitalisations during treatment

Nine patients (15%) required hospitalisation for toxicity; two patients for febrile neutropenia, one patient with fever and rigours without neutropenia, two patients with nausea and vomiting (including one patient with additional abdominal pain), one with diarrhoea (grade 3) and dehydration (grade 2), one for investigation of chest pain, one with porta-cath site erythema to investigate infection, and one with candidiasis and elevated blood sugar.

A total of 19 (31%) of patients were hospitalised for other reasons, not related to toxicity.

## DISCUSSION

The combination of oxaliplatin and 5-FU/LV has proven to be a major advance in the treatment of colorectal cancer both in the metastatic and now adjuvant setting (André *et al*, 2004). Studies with successive FOLFOX regimens are attempting to define the optimal oxaliplatin/5-FU/LV dose schedule. Important considerations include the haematological toxicity, a recognised side effect of both oxaliplatin and 5-FU treatment, and the cumulative neurotoxicity, which is dictated by oxaliplatin treatment duration.

In our study, we used a modified FOLFOX6 regimen, which focused upon maximising oxaliplatin dose intensity and improved patient convenience by allowing all patients to be treated as outpatients and reducing the number of outpatient visits. The response rate of 52% and the median PFS and OS of 8.2 and 18.7 months, respectively, is at the upper end of those reported in randomised trials of FOLFOX4 as front-line therapy, and similar to that of FOLFOX6 (Tournigand *et al*, 2004) in previously untreated metastatic colorectal cancer.

This is the first report of this modification to the FOLFOX6 regimen in untreated patients and the only report of this simplified FOLFOX regimen in either pretreated or untreated patients outside Europe. As such, it confirms a modification that is more convenient than FOLFOX4 and appears to have at least comparable, if not enhanced, efficacy, due to the increased oxaliplatin dose. In addition, there was neither a major increase in neurotoxicity or haematological toxicity compared to FOLFOX4 in the metastatic setting.

One of the two issues identified in the analysis of phase II studies of different FOLFOX combinations was the hypothesis that the dose intensity of oxaliplatin is important. Early studies with folfox 2 at 100 mg m^−2^ showed a high response rate (46%) but high neurotoxicity. Subsequently, reduction in oxaliplatin dose to 85 mg m^−2^ in Folfox4, lowered toxicity but compromised efficacy (24%), with a dose intensity of oxaliplatin of 74 mg m^−2^. (reviewed in Maindrault-Goebel *et al*, 2000). Thus, in FOLFOX6, the dose of oxaliplatin was increased from 85 to 100 mg m^−2^. However, in the phase II trials with this regimen, dose intensity remained reduced compared to Folfox2, where 89% of patients had >85 mg m^−2^ *vs* only 59% for Folfox6. In Folfox6 second line, the associated response rate also remained similar to Folfox4, possibly due to oxaliplatin dose modification for haematological toxicity (Maindrault-Goebel *et al*, 1999). This led to a redesigned schedule, FOLFOX7, which increased the dose of oxaliplatin further to 130 mg m^−2^, but reduced the total number of doses administered and focused on 5-FU dose reductions in response to non-neurological toxicity. In a phase II study in relapsed patients, FOLFOX7 produced a response rate of 42% and grade 3/4 neurological toxicity of 15% (Maindrault-Goebel *et al*, 2001). A subsequent study showed that a modified 5-FU schedule combined with 85 mg m^−2^ but with reductions of both oxaliplatin and 5-FU for haematologic toxicity once again reducing the oxaliplatin dose, was associated with marked reduction in efficacy in the second line setting – 12% in 37 patients but with a remarkable 72% response rate in 25 first-line patients (Cheeseman *et al*, 2002). The discrepancy remains unexplained, but still suggests that the need for enhanced dose intensity of oxaliplatin above 85 mg m^−2^ requires exploration.

In our study, we chose to focus on adjusting the 5-FU ahead of oxaliplatin and were able to achieve an impressive response rate and maintain oxaliplatin dose intensity similar to FOLFOX7 (85% with dose intensity >100 mg m^−2^ in first four cycles in Folfox7 Maindrault-Goebel *et al* (2001) *vs* 93% in ours), but without needing to increase the oxaliplatin dose further above 100 mg m^−2^. A potential benefit of our schedule lies in the issue of neurotoxicity.

Oxaliplatin-induced neurotoxicity consists of an acute rapid-onset sensory neuropathy, often triggered by cold or sudden temperature changes, and a chronic cumulative sensory neuropathy that occurs after several cycles of treatment, and is potentially dose limiting. In De Gramont's *et al* (2000) phase III study, 18% of patients experienced grade 3 sensory neurotoxicity; however, 74% of patients experienced a reversal of neurotoxicity after treatment was discontinued. In our study, 21% of patients experienced grade 3 neurotoxicity but with only a limited residual effect, as at 12 months 71% of patients had recovered. In the randomised FOLFOX6 study, 34% had grade 3 toxicity. Similarly, in the recent MOSAIC study (André *et al*, 2004), grade 3 neurotoxicity was observed in 12% of patients, with 94% of patients experiencing a partial or total recovery within 6 months of stopping treatment. This is summarised in Table 5 . Since the data suggest that cumulative dose, rather than dose intensity, leads to neurotoxicity, it follows that patients in our study would be allowed an increased number of treatment cycles compared with FOLFOX7.

An alternative approach to dealing with neurotoxicity is currently being tested by the French group in the Optimox studies (De Gramont *et al*, 2004), with previously untreated patients being randomised to FOLFOX4 until progression, or FOLFOX7 given for six cycles followed by a simplified LV5FU2 schedule every 2 weeks for 12 cycles. FOLFOX7 is then reintroduced for six cycles, or earlier in case of progression, in patients who have a response or SD at the first FOLFOX administration. From the preliminary results in 623 patients, the authors have concluded that the FOLFOX7 arm has a response rate and toxicity similar to the FOLFOX4 regimen and is more convenient. Only further follow-up will establish the role of shorter more intensive regimens *vs* the established FOLFOX approach. An intermediate regimen that maximises oxaliplatin dose intensity by focusing on 5-FU dose modification and preserving oxaliplatin dose may be equally valid.

The recent MOSAIC data (André *et al*, 2004) suggest that FOLFOX4 has a major role to play in the adjuvant setting, where enhanced efficacy with increased oxaliplatin dose intensity and improved convenience are even more important issues. The recent use of capecitabine combined with oxaliplatin improves convenience and has shown promising efficacy and tolerability data (Cassidy *et al*, 2004). This has led to a current randomised trial to examine if the capecitabine combination is equivalent to FOLFOX4, if it is, then attempts to increase oxaliplatin dose intensity in conjunction with capecitabine may be indicated. In the interim, this intermediate modification, with a higher dose of oxaliplatin and an entirely outpatient regimen, appears active, convenient and safe. Our focus on avoiding oxaliplatin dose reductions for haematological toxicity increased oxaliplatin dose intensity, and possibly improved efficacy, without unacceptable neurologic outcomes. As such, it may be recommended for use in the metastatic setting to enhance patient acceptability of this highly active treatment approach while awaiting evidence to support the routine use of capecitabine and the addition of targeted therapies such as bevacucimab and cetuximab.

## Figures and Tables

**Table 1:** Patient characteristics at registration (total 61 patients who received ⩾1 oxaliplatin dose)

	**No.**	**%**
*Sex*	45	74
Male	16	26
Female		
		
*ECOG performance status*		
0	29	48
1	24	39
2	8	13
		
*Primary site*		
Colon	40	66
Rectum	21	34
		
*Prior adjuvant therapy for colorectal cancer*		
No	41	67
Yes	20	33

**Table 2:** Best objective response to treatment

	**Total (56)**	
	**No.**	**%**
Complete response (CR)	3	5
Partial response (PR)	26	46
Stable disease (SD)	10	18
Progressive disease (PD)	9 (12)^a^	16 (21%)^a^
Not assessable (NA)^b^	8 (5)^a^	14 (9%)^a^

a Three patients were not assessable by our criteria, but displayed clinical disease progression that was not objectively validated (two received only two treatment cycles before clinical progression and one received four cycles and only one assessment before clinical progression).

b Not assessable patients were one patient who had clinical disease progression after two cycles but was too unwell to repeat imaging, five patients who received fewer than planned cycles of therapy, one patient whose scans were lost and was thus not assessed properly at cycle 7, and one patient who received only one assessment before clinical progression.

**Table 3:** Haematological toxicities (Worst grade while on treatment)

	**Total=61**		
	**Grade 2 – No. (%)**	**Grade 3 – No. (%)**	**Grade 4 – No. (%)**
Thrombocytopenia	9 (15)	4 (7)	0 (0)
Neutropenia	18 (30)	17 (28)	5 (8)
Febrile neutropenia	3 (5)		

**Table 4:** Nonhaematological toxicities

	**Total=61**		
	**Grade 2 – No. (%)**	**Grade 3 – No. (%)**	**Grade 4 – No. (%)**
Diarrhoea	12 (20)	7 (11)	0 (0)
Mucositis	7 (11)	2 (3)	0 (0)
Vomiting	7 (11)	2 (3)	0 (0)
Neurological	20 (33)	13 (21)	0 (0)
Fatigue	3 (5)	2 (3)	0 (0)
Nausea	6 (10)	2 (3)	0 (0)

**Table 5:** Correlation of oxaliplatin dose and dose intensity with outcomes (progression free survival and response rate) and toxicity

**Regimen (ref.)**	**Regimen no.**	**PFS mos**	**Planned dose mg m^−2^**	**Received dose intensity**	**Haematologic toxicity Grade 3/4 (%)**	**Neuro Tox. Grade 3 (%)**	**Resp. rate (%)**
Folfox *N*=46 (Maindrault-Goebelt *et al*, 2000)	2	7	100	0.98	39	33	46*
Folfox *N*=40 (Maindrault-Goebelt *et al*, 2000)	3	6	85	0.79	15	28	20*
Folfox *N*=57 (Andre *et al*, 2003)	4	5.1	85	0.89	37	16	24*
Folfox *N*=210 (De Gramont *et al*, 2000)	4	9.0	85	0.86	42	18	49*
Folfox *N*=60 (Maindrault-Goebelt *et al*, 2000)	6	5.3	100	0.86	24	16	27*
Folfox *N*=111 (Tournigand *et al*, 2004)	6	8.0	100	0.85	44	34	54*
Folfox *N*=48 (Maindrault-Goebelt *et al*, 2000)	7		130	0.85	9	27	42*
Cheeseman *N*=25 (Cheeseman *et al*, 2002)		10.6	85	NS	4	0†	72*
Goldstein *N*=61		8.2	100	0.83	36	21	51*

* =Second line Therapy;

† =Only assessed for first six cycles so not comparable.
